# Biogenic selenium nanoparticles synthesized by *Stenotrophomonas maltophilia* SeITE02 loose antibacterial and antibiofilm efficacy as a result of the progressive alteration of their organic coating layer

**DOI:** 10.1111/1751-7915.13260

**Published:** 2018-04-10

**Authors:** Eleonora Cremonini, Marzia Boaretti, Ilse Vandecandelaere, Emanuele Zonaro, Tom Coenye, Maria M. Lleo, Silvia Lampis, Giovanni Vallini

**Affiliations:** ^1^ Department of Diagnostic and Public Health University of Verona Strada Le Grazie 8 37134 Verona Italy; ^2^ Laboratory of Pharmaceutical Microbiology Ghent University Ottergemsesteenweg 460 9000 Gent Belgium; ^3^ Department of Biotechnology University of Verona Strada Le Grazie 15 37134 Verona Italy

## Abstract

Increasing emergence of drug‐resistant microorganisms poses a great concern to clinicians; thus, new active products are urgently required to treat a number of infectious disease cases. Different metallic and metalloid nanoparticles have so far been reported as possessing antimicrobial properties and proposed as a possible alternative therapy against resistant pathogenic microorganisms. In this study, selenium nanoparticles (SeNPs) synthesized by the environmental bacterial isolate *Stenotrophomonas maltophilia* SeITE02 were shown to exert a clear antimicrobial and antibiofilm activity against different pathogenic bacteria, either reference strains or clinical isolates. Antimicrobial and antibiofilm capacity seems to be strictly linked to the organic cap surrounding biogenic nanoparticles, although the actual role played by this coating layer in the biocidal action remains still undefined. Nevertheless, evidence has been gained that the progressive loss in protein and carbohydrate content of the organic cap determines a decrease in nanoparticle stability. This leads to an alteration of size and electrical properties of SeNPs along with a gradual attenuation of their antibacterial efficacy. Denaturation of the coating layer was proved even to have a negative effect on the antibiofilm activity of these nanoparticles. The pronounced antimicrobial efficacy of biogenic SeNPs compared to the denatured ones can – in first instance – be associated with their smaller dimensions. This study showed that the native organic coating layer of biogenic SeNPs functions in avoiding aggregation and maintaining electrostatic stability of the nanoparticles, thus allowing them to maintain efficient antimicrobial and antibiofilm capabilities.

## Introduction

In the last decades, widespread antibiotic treatment of clinical cases due to bacterial and fungal infections has become a threat because of increasing occurrence of antimicrobial resistance (AMR) within microorganisms that are often able to form fastidious biofilms on tissues and medical devices (Penesyan *et al*., 2015). Both aspects represent a serious concern as available antimicrobial drugs have proved to be poorly active or inefficacious giving rise to the emergence of chronic infections and an increase in morbidity and death rate. This has led to a growing awareness that new approaches, including those based on the use of non‐antibiotic antibacterial agents, need to be perfected to face the antibiotic resistance challenge (Beyth *et al*., 2015).

In this perspective, tailored metal nanoparticles (NPs) have recently come to the forefront as promising antibacterial and antibiofilm agents (Rizzello and Pompa, 2014; Shakibaie *et al*., 2015). For instance, silver nanoparticles (AgNPs), one of the most studied classes of NPs, have been extensively considered in the last two decades as antimicrobial and anticancer agent drug delivery systems as well as in diagnostics and probing for the treatment of various diseases (Yuan *et al*., 2017). AgNPs are likely to exert their antibacterial effects in a dose‐ and time‐dependent manner. Moreover, silver nanoparticles have been demonstrated able to perform their antibacterial activity through the generation of reactive oxygen species (ROS), production of malondialdehyde (MDA), and leakage induction of proteins and sugars from bacterial cells (Yuan *et al*., 2017). Although the mechanisms underlying the antibacterial effect of metal NPs have not yet been elucidated, it is generally believed that induction of oxidative stress, release of metal ions and/or non‐oxidative reactions are involved (Wang *et al*., 2017). According to the information available from previous investigations, the major processes by which NPs exert antibacterial activity can be summarized as follows: (i) penetration into and disruption of bacterial cell membrane, (ii) generation of ROS and (iii) induction of harmful intracellular reactions, including damages on DNA and proteins (Wang *et al*., 2017).

Recently, Cremonini *et al*. (2016) reported on comparative tests carried out with selenium nanoparticles (SeNPs) of biogenic nature, produced by two environmental bacterial strains [namely, *Bacillus mycoides* SeITE01 and *Stenotrophomonas maltophilia* (SeITE02) and chemically synthesized selenium nanoparticles (Ch‐SeNPS)]. Biogenic SeNPs showed a higher antibacterial activity against a number of clinical strains of *Pseudomonas aeruginosa* as well as a significant inhibition of biofilm formation along with disaggregation capacity towards already established bacterial biofilms. Furthermore, biogenic SeNPs showed no toxic effects in cell cultures of human dendritic cell (DCs) or fibroblasts and did not elicit production of pro‐inflammatory and immune‐stimulatory cytokines (Cremonini *et al*., 2016).

By relying on these results, the aim of this study was to investigate a possible wider antimicrobial activity of SeNPs from *S. maltophilia* SeITE02 against an extended list of bacterial species and strains, with attention to specific factors that could play a role in modulating the efficacy of such nanoparticles. In particular, we focused on the possible variations in antimicrobial and antibiofilm capacity of SeNPs as a consequence of the denaturation of their organic coating layer.

## Results

### Biosynthesis and structural features of selenium nanoparticles

As reported in previous studies (Jain *et al*., 2015; Cremonini *et al*., 2016; Lampis *et al*., 2017), biogenic SeNPs from the bacterial strain *Stenotrophomonas maltophilia* SeITE02 present a complex organic cap consisting of different biomolecules such as proteins, lipids and carbohydrates. In order to evaluate the possible influence of such an organic coating layer on the biological reactivity of these biogenic SeNPs (here called SeNPs‐24 and also untreated SeNPs), different and progressively more aggressive denaturants were applied to the nanoparticles. Quantification of surface‐associated macromolecular matrix as well as measurement of physical and electrical properties was performed on untreated and differently treated SeNPs.

**Figure 1:**
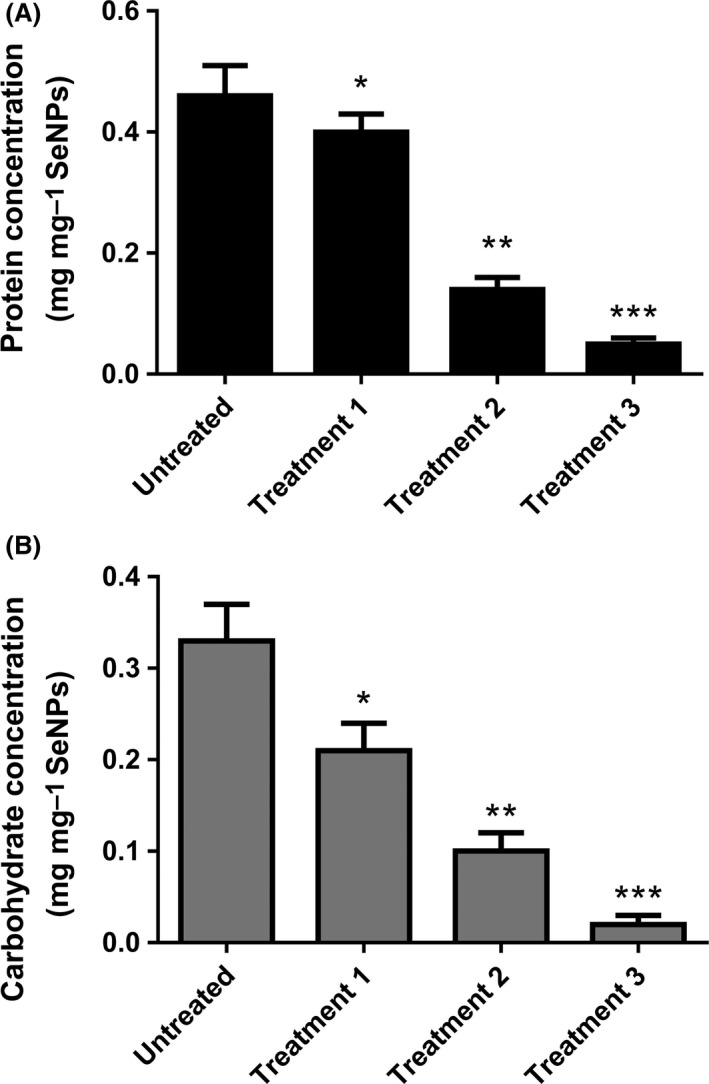
Protein (A) and carbohydrate (B) content of SeNPs as biogenic product and after different treatments (*n* = 3; *P* < 0.05). Untreated: biogenic SeNPs; Treatment 1: 10% SDS; Treatment 2: 10% SDS+10 min boiling; Treatment 3: 10% SDS+30 min boiling.

Figure 1A shows how the protein content associated with the organic coating layer of SeNPs decreases after exposure to the different denaturing agents. The concentration of proteins associated with untreated biogenic SeNPs is 0.46 ± 0.05 mg mg^−1^ NPs; this value progressively decreases to 0.05 ± 0.01 mg mg^−1^ NPs after exposure to the most drastic denaturation by means of 10% sodium dodecyl sulphate (SDS) and 30‐min boiling. A similar pattern was observed for carbohydrate concentration (Fig. 1B) with untreated biogenic SeNPs showing a value of 0.33 ± 0.04 mg mg^−1^ NPs that then fell to 0.02 ± 0.01 mg mg^−1^ NPs after the strongest denaturing treatment.

Dynamic light scattering measurements (Table 1) showed an average size for untreated biogenic SeNPs of 181 ± 20 nm with a Z‐potential value of −32.01 mV. Exposure to different denaturing treatments caused a progressive growth of nanoparticle dimensions, up to 270 ± 24 nm after the treatment with 10% SDS and 30‐min boiling. In particular, as shown in Fig. S1, the progressively more denaturing treatment of biogenic NPs resulted in an increase in the number of SeNPs of larger size and more susceptible to aggregate each other. On the other hand, values of the Z‐potential of SeNPs ranged within −17.40 and −3.97 mV after different denaturing procedures (Table 1).

**Table 1:** Dynamic light scattering analysis and zeta potential of SeNPs as biogenic product and after different treatments (*n* = 3; average ± SD). Untreated: biogenic SeNPs; Treatment 1: 10% SDS; Treatment 2: 10% SDS + 10 min boiling; Treatment 3: 10% SDS + 30 min boiling

	Diameter (nm)	Ζ‐potential (mV)
Untreated SeNPs	181 ± 20	−32.01 ± 2.67
Treatment 1	209 ± 25	−17.40 ± 3.45
Treatment 2	233 ± 19	−8.11 ± 2.36
Treatment 3	270 ± 24	−3.97 ± 1.34

Another type of SeNPs (SeNPs‐48) was also taken into consideration: these SeNPs were obtained from bacterial cultures after 48 h of incubation with sodium selenite and showed a diameter of 276 ± 26 nm, with a Z‐potential value of −29.27 mV (Fig. S2A and B). The protein concentration associated with these biogenic nanostructures was 0.48 ± 0.07 mg mg^−1^ SeNPs, while the carbohydrate concentration was 0.35 ± 0.03 mg mg^−1^ SeNPs (Fig. S2C).

### Antimicrobial activity of SeNPs in different conformational states

A number of bacterial strains belonging to different species were screened for the susceptibility to untreated and treated SeNPs. These bacterial species/strains were selected based on their actual occurrence in biofilm‐mediated infections. Therefore, *Pseudomonas aeruginosa* and *Burkholderia cenocepacia* as well as emerging harmful pathogens such as *Achromobacter xylosoxidans* and *Stenotrophomonas maltophilia* causing chronic lung infections in cystic fibrosis and immunodepressed patients (Bjarnsholt, 2013) were taken into account. On the other hand, as far as Gram‐positive bacterial agents are concerned, staphylococci were considered important producers of biofilms associated with the implantation of biomedical devices or skin pathologies (Paharik and Horswill, 2016).

As shown in Table 2, MIC values of biogenic SeNPs varied widely among the different microbial species and even diverse strains belonging to the same species, ranging from 4 to 128 μg ml^−1^. Nevertheless, an evaluation of the activity of SeNPs on the different bacterial strains tested (in terms of susceptibility or resistance) turned out to be quite difficult as standard break points do not exist and a comparison with the susceptibility to antibiotics is not predictable. Two groups of strains could be distinguished: one showing low values of MIC (4–16 μg ml^−1^) while the other showing higher MIC values (32–128 μg ml^−1^), although a strict association with specific bacterial species or even families cannot be done. For instance among *P. aeruginosa* strains, PAO1 and BR2 showed low values of MIC while in INT and BR1 higher MIC values were measured. The same was also demonstrated for *Staphylococcus aureus,* Mu50 strain showing the highest MIC value of 128 μg ml^−1^. As a general rule, it seems that Gram‐positive strains responded to SeNPs with lower MIC values when compared to the Gram‐negative ones. Anyway, the number of bacterial strains studied so far is too restricted to confirm such a trend. To evaluate the influence by the whole organic cap of biogenic selenium nanoparticles on their antimicrobial efficacy, such an activity was tested with nanoparticles subjected to progressively stronger protocols for the denaturation of the external organic coating. Data reported in Table 2 show, for almost all the strains screened, a decrease in the antimicrobial activity of SeNPs, which experienced the most intensive denaturation, as revealed by progressively higher MIC values.

**Table 2:** Minimum inhibitory concentration (MIC) of different types of SeNPs against various bacterial strains. Untreated: biogenic SeNPs; Treatment 1: 10% SDS; Treatment 2: 10% SDS + 10 min boiling; Treatment 3: 10% SDS + 30 min boiling. Data are expressed as the average of three biological replicates

Bacterial species	Strain name	MIC μg ml^−1^			
		Untreated SeNPs	Treatment 1	Treatment 2	Treatment 3
*P. aeruginosa*	PAO1	8	16	128	64
*P. aeruginosa*	INT	64	512	> 512	> 512
*P. aeruginosa*	BR1	32	64	32	64
*P. aeruginosa*	BR2	8	16	32	128
*S. maltophilia*	VR10	32	16	32	64
*S. maltophilia*	VR20	64	256	512	> 512
*A. xylosoxidans*	C	64	> 512	> 512	> 512
*B. cenocepacia*	LMG16656	16	16	32	32
*S. aureus*	Mu50	128	128	256	512
*S. aureus*	UR1	4	16	32	64
*S. haemolyticus*	UST1	16	8	32	64
*S. epidermidis*	ET024	4	8	16	64

To evaluate the bioactivity of SeNPs still surrounded by an intact organic cap with those of similar size but extensively denatured, SeNPs‐48 were tested against those strains that had shown the highest MIC values against denatured SeNPs, namely *P. aeruginosa* PAO1, *P. aeruginosa* BR2, *S. maltophilia* VR20 and *S. aureus* UR1. As reported in Fig. S2D, the MIC values for SeNPs‐48 were lower than those registered with most extensively denatured SeNPs, ranging between 16 and 256 μg ml^−1^ with reference to the different bacterial strains tested.

### Antibiofilm activity of SeNPs in different conformational states

Efficacy of both untreated and denatured SeNPs in preventing bacterial biofilm formation or promoting biofilm eradication was also evaluated. Tests were carried out on a selection of bacterial strains, namely PAO1, BR1 and BR2 of *P*. *aeruginosa*,* B. cenocepacia* and the *S. haemolitycus*. The choice was made on the basis of low MIC values for SeNPs shown by the respective planktonic cultures and the high capacity of biofilm formation of these strains. On the other hand, *S. aureus* Mu50 showing methicillin resistance as well as being known as moderately resistant to vancomycin was also taken into consideration as a bacterial strain able to withstand the presence of antibiotics and SeNPs. By relying on the results of preliminary tests (data not shown), antibiofilm activity of SeNPs in different conformational states was measured at a nanoparticle concentration of 128 μg ml^−1^, found as the lowest concentration with a significant contrasting effect on biofilm formation.

**Figure 2:**
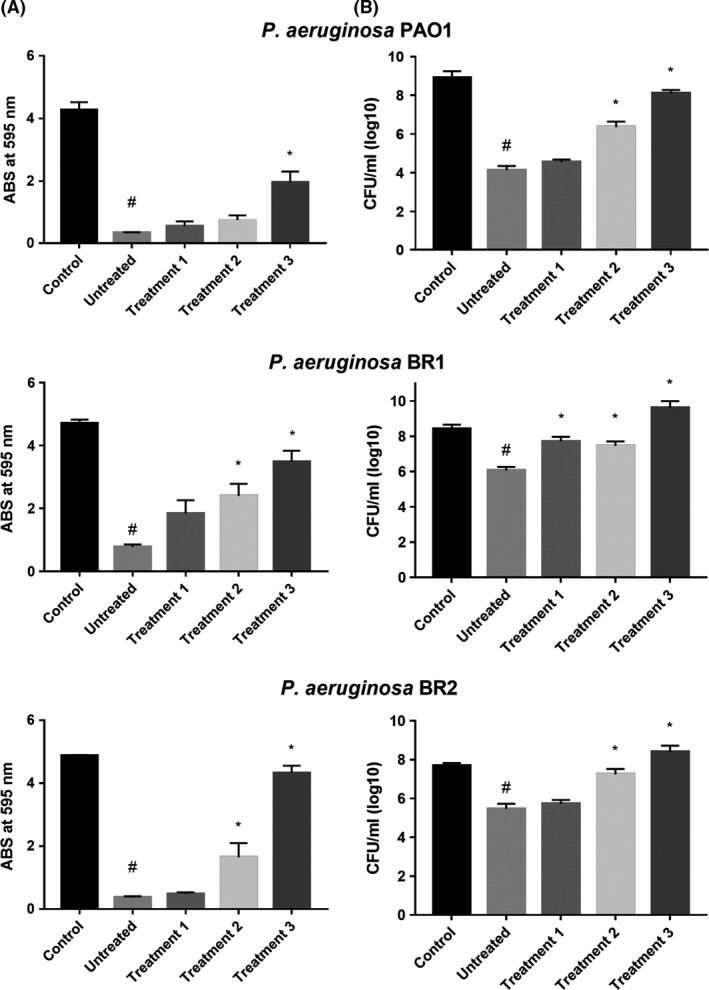
Antibiofilm activity of different types of SeNPs measured by CV staining (effect on biofilm biomass) (A) and colony counting (effect on cell viability) (B), as regards *P. aeruginosa* strains PAO1, BR1 and BR2 (*n* = 3, Average ± SEM). #*P* < 0.05 compared to a biofilm grown in the absence of SeNPs (Control); *P < 0.05 compared to a biofilm treated with ‘untreated SeNPs’. Untreated: biogenic SeNPs, Treatment 1: 10% SDS, Treatment 2: 10% SDS + 10 min boiling, Treatment 3: 10% SDS+30 min boiling.

**Figure 3:**
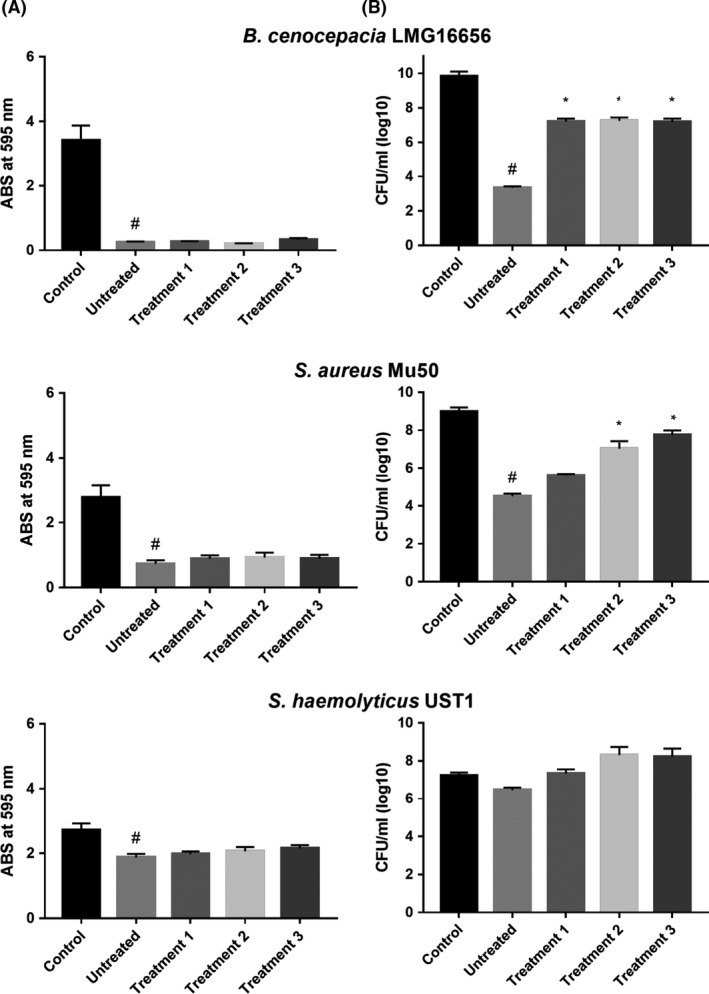
Antibiofilm activity of different types of SeNPs measured by CV staining (effect on biofilm biomass) (A) and colony counting (effect on cell viability) (B), as regards *B. cenocepacia* LMG 16656, *S. aureus* Mu50 and *S. haemoliticus* UST1 (*n* = 3, Average ± SEM). # *P* < 0.05 compared to a biofilm grown in the absence of SeNPs (Control); * *P* < 0.05 compared to a biofilm treated with ‘untreated SeNPs’. Untreated: biogenic SeNPs, Treatment 1: 10% SDS, Treatment 2: 10% SDS + 10 min boiling, Treatment 3: 10% SDS + 30 min boiling.

Quantification of the total biofilm biomass singularly formed by the six bacterial strains tested was obtained through crystal violet (CV) staining. Afterwards, counts of viable cells (reported as CFU ml^−1^) still present within biofilm matrices were determined by plating aliquots of biofilms on an agarized growth substrate. Exposure of biofilms produced by all *P. aeruginosa* strains tested to 128 μg ml^−1^ of SeNPs in their different conformational states resulted in a clear decrease in the CV signal. This effect was more pronounced with untreated biogenic SeNPs rather than with most extensively denatured SeNPs (Fig. 2A). Culturable cells of *P. aeruginosa* PAO1 decreased significantly after treatments with different types of SeNPs with the exception of those harshly denatured (Fig. 2B). On the other hand, the number of CFU ml^−1^ counted from biofilms of *P. aeruginosa* BR1 and BR2 decreased significantly only in case of exposure to untreated SeNPs (Fig. 2B). Moreover, it is worth noting that the biofilm generated by *B. cenocepacia* LMG 16656 was quite susceptible to degradation by all conformational types of SeNPs, including most extensively denatured nanoparticles. Numbers of culturable cells recovered from biofilms of this strain, exposed to all kind of SeNPs, were significantly lower than those from control biofilms (Fig. 3).

As far as strain Mu50 of *S. aureus* is concerned, despite the low susceptibility to the action of SeNPs in planktonic state (MIC of 128 μg ml^−1^), its biofilm depositions were easily degradable by all forms of SeNPs tested. A decrease in the number of culturable cells associated with the biofilm matrix was observed after treatment with selenium nanoparticles. The lowest effects were, however, revealed when most denatured SeNPs were applied. Finally, *S. haemolyticus* UST1 that synthesizes a very SeNP‐resistant biofilm did not show any decrease in the number of culturable cells after exposure to all the NPs tested (Fig. 3).

The comparison of the effects on biofilm biomass and cell viability between the different types of SeNPs tested (Figs 2 and 3) showed a difference not only in terms of the control but also among the various denaturing treatments applied to the NPs. In all the strains tested, except for the *S. haemolyticus* UST1 strain, the progressive denaturation of the surface biomolecular cap is related to a loss of their antibiofilm activity shown by an increasing value of CV signal and increasing number of CFU ml^−1^. The statistic analysis reported, indeed, points out a significant difference in particular between the antibiofilm activity of the untreated NPs and the ones exposed to the harshly denaturing treatment.

### Role of reactive oxygen species induced by SeNPs

**Figure 4:**
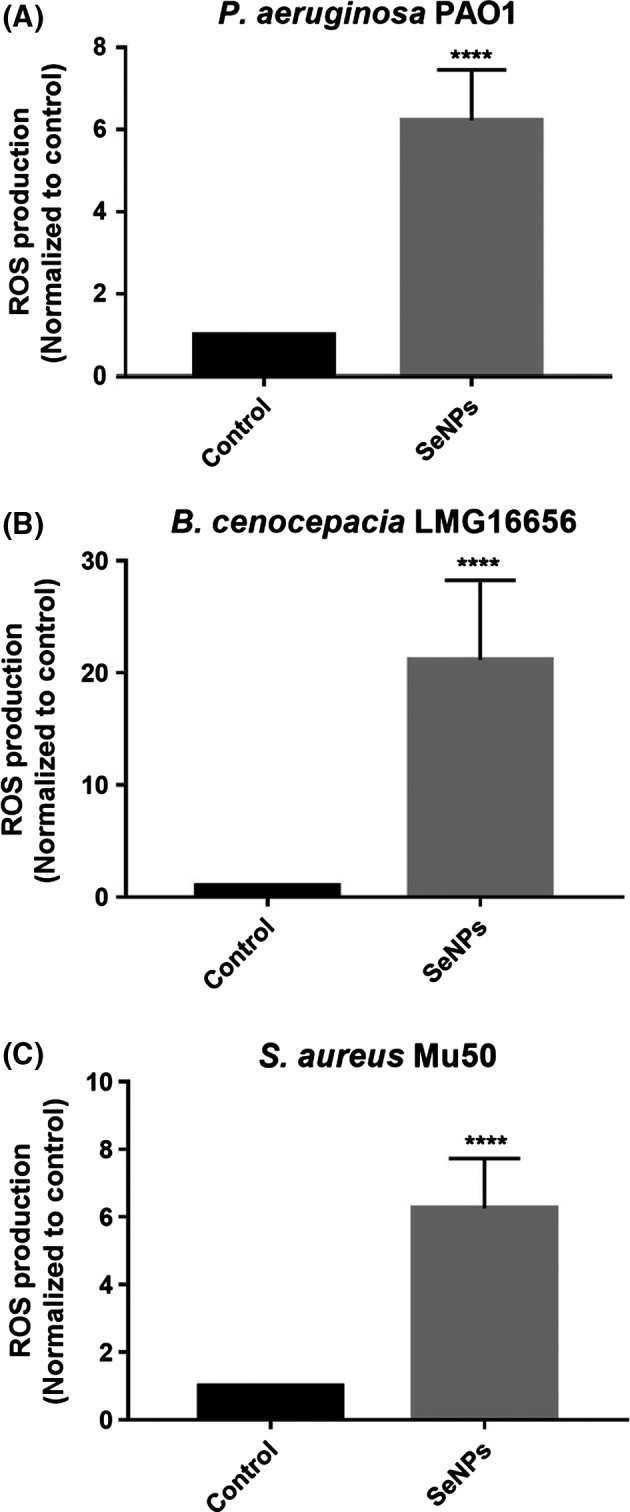
Formation of ROS in *P. aeruginosa, B. cenocepacia, S. aureus* cells exposed to SeNPs for 24 h (SeNPs) normalized to cells not exposed to NPs (Control) (*n* = 3; average ± SEM;* P* < 0.05).

Reactive oxygen species (ROS) are important elements in the bacterial response to physical and chemical environmental stresses and have been associated with the killing action of a variety of antimicrobial agents (Van Acker and Coenye, 2017). The production of ROS in response to treatment with biogenic SeNPs was analysed in *P*. *aeruginosa* PAO1, *S*. *aureus* Mu50 and *B*. *cenocepacia* LMG16656 strains. As shown in Figure 4, in all bacterial strains tested an increase in ROS produced after treatment with biogenic nanoparticles was observed compared to the untreated controls.

## Discussion

Over the last two decades, several authors have made increasing efforts to develop innovative antimicrobial agents based on metal/metalloid nanostructured particles as an alternative strategy to overcome the worrisome increase in antibiotic resistance. Antibiotic‐resistant infections are in fact more and more widespread, particularly in nosocomial contexts. The emergence of such pathogenic conditions generates in turn further risks due to the recourse to higher doses of drugs or additional toxic therapeutic treatments which can be responsible for longer hospital stays and an increased death rate (Ferri *et al*., 2015). Major concerns are associated in this respect with multidrug resistance (MDR) bacteria and biofilm‐forming microbial communities.

In this scenario, selenium nanoparticles either of biogenic origin or chemically synthesized have been proven to possess surprising antibacterial and antibiofilm capabilities (Cihalova *et al*., 2015; Cremonini *et al*., 2016; Huang *et al*., 2016).

The present study focused on the influence that the outer organic coating layer of biogenic SeNPs synthesized by the bacterial strain *S. maltophilia* SeITE02 (Zonaro *et al*., 2015; Lampis *et al*., 2017) plays in determining antimicrobial and antibiofilm efficacy of such nanostructured particles. Actually, biogenic SeNPs produced by the strain SeITE02 are surrounded by an organic cap mainly consisting of proteins, lipids and carbohydrates (Cremonini *et al*., 2016; Lampis *et al*., 2017). Four different types of biogenic elemental selenium nanoparticles obtained as a result of 24‐h incubation of *S. maltophilia* SeITE02 in the presence of selenite were considered according to the follow scheme: (i) untreated SeNPs as well as the same biogenic nanoparticles subjected, however, to increasingly stronger denaturing procedures, namely (ii) denatured through a treatment with 10% SDS, (iii) treated with 10% SDS and 10‐min boiling, and (iv) strongly denatured with 10% SDS and 30‐min boiling. By increasing the strength of denaturing treatments, a progressive loss of proteins and carbohydrates from the organic coat surrounding SeNPs was shown along with an increment in nanoparticle size and a change in their Z‐potential.

Growth in size of biogenic selenium nanoparticles as a consequence of denaturation due to partial loss of the external coating layer was an expected result as this phenomenon is widely reported in the literature (Dobias *et al*., 2011). Therefore, the presence of an organic cap surrounding biogenic SeNPs confirmed its pivotal role in preventing particle aggregation. Furthermore, the shift of Z‐potential measurements towards less negative values also reduced the stability of untreated SeNPs. It is in fact well known that NPs with charges close to a neutral value tend to coalesce (Hunter, 1981). Moreover, antimicrobial activity of each type of SeNPs tested against different bacterial pathogens was quantified by MIC value determination. Results showed a wide variability of bacterial response to distinct kind of SeNPs as regards this parameter not only among different species but also within strains belonging to the same species. Despite this variable antimicrobial efficacy of untreated SeNPs towards the different bacterial strains tested, as a general rule MIC values increased with the progressive denaturation of the organic coating layer. These findings suggest that antimicrobial activity of SeNPs is size‐dependent, with higher inhibiting effects associated with the smallest ones, as already reported in previous studies (Lu *et al*., 2013; Chudobova *et al*., 2014; Zonaro *et al*., 2015). Indeed, denaturation of the external organic cap which leads to a progressive increase in particle dimensions coincides with a gradual drop in antibacterial efficacy. Nevertheless, also variations in the qualitative and quantitative features of the organic coating seem to affect the antimicrobial properties of SeNPs biosynthesized by *S. maltophilia* SeITE02. To verify a direct influence of the organic layer surrounding these nanoparticles on their biocidal potential, SeNPs from strain SeITE02 cultures after a 48‐h incubation (SeNPs‐48), larger in size than those recovered from a 24‐h culture (SeNPs‐24) but with similar size to the strongly denatured ones, were tested against the bacterial strains that have shown to withstand the highest MIC values. Interestingly, these biogenic SeNPs‐48 owning their native surrounding layer showed MIC values significantly lower than those detected with more extensively denatured SeNPs. Therefore, based on these observations, it can be argued that the organic cap surrounding biogenic SeNPs plays a direct role in the efficacious antimicrobial interaction with target bacterial planktonic cells and biofilms.

In conclusion, factors that are likely to concur in influencing the antimicrobial potential of biogenic metal/metalloid nanoparticles can be summarized as follows: (i) the nature of constitutive elements (Natan and Banin, 2017); (ii) the size (Lu *et al*., 2013; Zonaro *et al*., 2015) and (iii) the surface architecture (Verma and Stellacci, 2010). Much is still to be clarified, however, in order to elucidate in detail the nature of the external organic coating of biogenic metal nanoparticles for an actual interpretation of the intimate biocidal mechanisms.

Finally, in order to investigate in depth the antibiofilm activity of SeNPs already highlighted in previous findings by the authors (Cremonini *et al*., 2016), the impact on either biofilm biomass or viability of bacterial cells embedded in biofilm extracellular polymeric substances (EPS) was evaluated. With reference to the induction of consistency loss of biofilm biomass, untreated SeNPs were able to significantly disrupt the EPS matrix produced by all bacterial strains tested. Moreover, quantification of culturable cells trapped inside bacterial biofilms confirmed the biocidal potential of untreated SeNPs, with a marked reduction in counts in five of the six strains tested, with the exception of *S. haemolyticus* UST1, which instead retained cell viability. Results from the tests carried out with differently denatured SeNPs, compared to the untreated nanoparticles, highlighted a progressively weaker effect on biofilm with a strength increase in denaturation treatments, confirming the key role played by the organic cap even in terms of antibiofilm activity.

Although mechanisms through which metal/metalloid nanoparticles exert antimicrobial activity are not completely understood, a number of authors claim the production of reactive oxygen species (ROS) as one of the possible modes of action (Manke *et al*., 2013; Yan *et al*., 2013). In particular, antimicrobial effects of different selenium compounds have been attributed to the formation of free radicals (Tran *et al*., 2009). In this study, exposure of three bacterial strains (namely *P*. *aeruginosa* PAO1, *S*. *aureus* Mu50 and *B*. *cenocepacia* LMG16656) to untreated SeNPs actually caused an increase in ROS production compared to the controls. The amount of ROS observed in our experiments is in the same order of magnitude as those that were induced by some antibiotics (Van Acker *et al*., 2016) or by selenite alone (Zonaro *et al*., 2015).

In conclusion, with the present investigation, evidence has been found that biogenic SeNPs, synthesized by an eco‐friendly process can reasonably be considered a reliable antimicrobial and antibiofilm agent capable of efficaciously inhibiting fastidious biofilm‐producing bacteria of medical concern. Despite the formation of ROS, which could contribute to the antiimicrobial activity of SeNPs, features of the organic coat surrounding biogenic SeNPs were correlated to a marked influence on the antimicrobial properties of those nanoparticles. The external coating layer seems in fact to have a pivotal role in hindering particle aggregation, maintaining nanoparticles in a functional conformational state and favouring their interaction with bacterial cells.

The information gained so far on biogenic SeNPs opens a realistic perspective for a possible use of these nanostructured particles as a novel non‐antibiotic antimicrobial tool to treat challenging nosocomial infections, including biofilm‐associated syndromes and diseased states caused by multidrug‐resistant bacteria. Further investigations are, however, required to elucidate in detail the actual mechanisms of action of these nanoparticles as well to evaluate their whole biological compatibility with the human body.

## Experimental procedures

### Biosynthesis of biogenic SeNPs

Selenium nanoparticles were produced and purified from *Stenotrophomonas maltophilia* SeITE02 culture after 24 h of growth in Nutrient Broth supplied with 0.5 mM Na_2_SeO_3_.

The microbial culture was incubated in the dark at 27°C on a rotary shaker at 200 r.p.m. Bacterial cells and SeNPs were removed from culture medium after 24 h by centrifuging at 10 000 × *g* for 10 min. The pellets were washed twice with 0.9% NaCl solution, resuspended in Tris/HCl buffer (pH 8.2), and cells were then disrupted by ultrasonication at 100 W for 5 min. The suspension was centrifuged at 10 000 × *g* for 30 min to separate disrupted cells (pellet) from SeNPs (supernatant). SeNPs were recovered after centrifugation at 40 000 × *g* for 30 min, washed twice and resuspended in deionized water (Zonaro *et al*., 2015; Cremonini *et al*., 2016). The SeNPs thus synthesized were indicated as SeNPs‐24 and also as ‘untreated SeNPs’. The same experimental protocol of preparation was applied using a microbial culture incubated in the presence of 0.5 mM sodium selenite for 48 h. The SeNPs obtained were indicated as SeNPs‐48.

### Nanoparticle treatments and quantification of proteins and carbohydrates

Biogenic SeNPs were collected through centrifugation at 16 000 r.p.m. and subsequently exposed to three different treatments: 10% SDS (treatment 1); 10% SDS and boiling for 10 min (treatment 2); 10% SDS and boiling for 30 min (treatment 3) (Dobias *et al*., 2011). SeNPs were then centrifuged at 16 000 r.p.m., and the supernatants were separated to quantify protein and carbohydrate content obtained after different treatments. The evaluation of proteins and carbohydrates was applied also on untreated SeNPs and SeNPs‐48. Protein concentration was determined following the method of Lowry *et al*. (1951) using bovine serum albumin (BSA) as standard; carbohydrates were measured using the anthrone method (Roe, 1955) using glucose as standard. Differences between the protein and carbohydrate content after treatments were determined using one‐way analysis of variance (ANOVA) with graphpad prism 6.0 (GraphPad Software Inc., La Jolla, CA, USA). The level of significance was set at *P* < 0.05. All tests were carried out in triplicate (*n* = 3), and the results were averaged.

SeNPs‐24, SeNPs‐48 and SeNPs obtained after different treatments were characterized by means of dynamic light scattering (DLS) analysis. DLS was carried out using a Zen 3600 Zetasizer Nano ZS (Malvern Instruments, Malvern, UK) equipped with a 633‐nm helium–neon laser light source (4.0 mW), detecting scattering information at a fixed angle of 173°. SeNP samples (300 μl) were transferred to a quartz cuvette (10 mm path length), and the mean size distribution and zeta potential were recorded at 25°C using the software provided by Malvern Instruments.

### Microbial strains and growth conditions

Experiments were conducted with both reference and clinical strains. Specifically, we analysed four strains of *Pseudomonas aeruginosa,* namely *P. aeruginosa* PAO1 (reference strain), INT (multi‐resistant clinical isolate) and BR1 and BR2 (both isolated from bronchial aspirates), two clinical strains of *Stenotrophomonas maltophilia* (*S. maltophilia* VR10 and VR20), *Achromobacter xyloxidans* strain C and *Burkholderia cenocepacia* strain LMG 16656. As far as Gram‐positive bacterial strains, we included in the study the methicillin‐resistant *Staphylococcus aureus* Mu50 strain (reference strain) and a clinical strain isolated from an urine sample (*S. aureus* UR1), as well as *Staphylococcus epidermidis* ET024, isolated from a biofilm on an endotracheal tube (Vandecandelaere *et al*., 2014) and *Staphylococcus haemolitycus* UST1, a clinical isolate from a burn wound. All bacterial strains were grown in Tryptone Soy Broth (Oxoid, Basingstoke, England) at 37°C.

### Determination of the minimum inhibitory concentration (MIC) of biogenic NPs

The susceptibility of each strain to different types of SeNPs was determined in triplicate according to the National Committee for Clinical and Laboratory Standards (NCCLS) protocol using the broth microdilution method in flat‐bottom 96‐well microtiter plates. The microbial inoculum was standardized to approximately 10^5^ CFU ml^−1^. Plates were incubated at 37°C for 24 h, and the optical density (O.D.) at 590 nm was determined using a multilabel microtiter plate reader (Envision, Perkin‐Elmer LAS, Waltham, MA, USA). The MIC was recorded as the lowest SeNP concentration at which no significant O.D. increase was observed.

### Biofilm formation and treatment

Polystyrene round‐bottomed 96‐well microtiter plates were inoculated with 100 μl of a bacterial suspension containing approximately 5 × 10^7^ CFU ml^−1^ and incubated at 37°C for 4 h. The biofilms formed were rinsed once with 100 μl of physiological saline solution (PS) to remove all non‐adherent cells and subsequently treated with 128 μg ml^−1^ of the four different kinds of SeNPs diluted in PS. For every strain, untreated biofilms were included as controls. Then, 100 μl of fresh medium was added to each well and the plates were incubated for an additional 20 h at 37°C (Peeters *et al*., 2008).

### Quantification of the biofilm biomass (CV assay)

After 24 h of incubation, biofilms were rinsed with 100 μl PS and were fixed by addition of 100 μl 99% methanol. After an incubation of 15 min at room temperature, the supernatant was removed and the plates were air‐dried at 37°C. Then, 100 μl of a 0.1% CV solution was added to each well (20‐min incubation at room temperature). The dye‐stained total biofilm mass included live, dead cells and extracellular polymeric substances (EPS) grown on the bottom and the walls of microtiter plate wells. The excess of CV was removed by washing the plate under running tap water. Finally, 150 μl of 33% acetic acid was added to resolubilize the stain and the plate was put on a vortex for at least 20 min (800 r.p.m.). The attached bacterial biomass was measured by evaluation of absorbance at 590 nm (Peeters *et al*., 2008).

### Quantification of surviving cells

After 24 h of incubation, cells were collected by two cycles of sonication and vortexing. The cell pellet obtained after centrifugation (5 min at 13 000 r.p.m.) was suspended in 1 ml of PS, and the number of colony‐forming units (CFU) was determined by plating on TSA (Tryptone Soy Agar, Oxoid) (Peeters *et al*., 2008).

### Measurement of ROS production

ROS production by bacterial cultures after treatment with biogenic nanoparticles was investigated. The concentration of SeNPs used corresponded to the MIC for all strains tested.

Overnight cultures of every strain were dispensed in tubes, and 2‐7‐dichlorodihydrofluorescein diacetate (H_2_DCF‐DA, Sigma‐Aldrich, Bornem, Belgium) was added at a final concentration of 10 μM. All tubes were incubated for 1 h at 37°C and then centrifuged for 5 min at 13 000 r.p.m. Biogenic NPs were added to two aliquots of cells (one with H2DCF‐DA and one without). Appropriate controls, to which an equal volume of PS was added instead of NPs, were also included. All cell suspensions were transferred to a black microtiter plate. Six wells were filled per condition. The fluorescence (λ_ex_ = 485 nm and λ_em_ = 535 nm) was measured every 30 min for approximately 24 h with microtiter plate reader (Perkin‐Elmer LAS). The net fluorescence emission by the NP‐treated and NP‐untreated cells was calculated, and a corresponding graph was created. The results are only comparable within a plate and not between different plates (Wang and Joseph, 1999).

### Statistical analysis

The data are expressed as means plus standard error meaning (SEM). Statistical analyses, including *t*‐test and one‐way analysis of variance (ANOVA), were performed using graphpad prism 6.0 (GraphPad Software Inc., La Jolla, CA, USA). All experiments were carried out in triplicate. The level of significance was set at *P* value <0.05.

## Conflict of interest

The authors have no conflict of interest to declare.

## Supporting information


**Fig. S1.** Diameter distribution of SeNPs extracted from *Stenotrophomonas maltophilia* SeITE02. Biogenic SeNPs (A), SeNPs after treatment with 10% SDS (B), SeNPs after treatment with 10% SDS +10 min boiling (C), SeNPs after treatment with 10% SDS + 30 min boiling (D).
**Fig. S2.** SeNPs‐48: dynamic light scattering analysis and zeta potential (A), diameter distribution (B), protein and carbohydrate concentrations (C), MIC (Minimum Inhibitory Concentration) values of SeNPs‐48 against various bacterial strains (D).
