# Supplementary material for: Biogenic selenium nanoparticles synthesized by Stenotrophomonas maltophilia SeITE02 loose antibacterial and antibiofilm efficacy as a result of the progressive alteration of their organic coating layer
Source: Microb Biotechnol. 2018 Apr 10;11(6):1037–47. doi: 10.1111/1751-7915.13260 (PMC6196382; doi:10.1111/1751-7915.13260)
Supplement: Supplementary file 1 — Fig. S1. Diameter distribution of SeNPs extracted from Stenotrophomonas maltophilia SeITE02. Biogenic SeNPs (A), SeNPs after treatment with 10% SDS (B), SeNPs after treatment with 10% SDS +10 min boiling (C), SeNPs after treatment with 10% SDS + 30 min boiling (D). Fig. S2. SeNPs‐48: dynamic light scattering analysis and zeta potential (A), diameter distribution (B), protein and carbohydrate concentrations (C), MIC (Minimum Inhibitory Concentration) values of SeNPs‐48 against various bacterial strains (D). [file MBT2-11-1037-s001.pdf]

Figure legends

Figure S1. Diameter distribution of SeNPs extracted from *Stenotrophomonas maltophilia* SeITE02. Biogenic SeNPs (A), SeNPs after treatment with 10% SDS (B), SeNPs after treatment with 10% SDS +10 min boiling (C), SeNPs after treatment with 10% SDS + 30 min boiling (D).

Figure S2. SeNPs-48: dynamic light scattering analysis and zeta potential (A), diameter distribution (B), protein and carbohydrate concentrations (C), MIC (Minimum Inhibitory Concentration) values of SeNPs-48 against various bacterial strains (D).

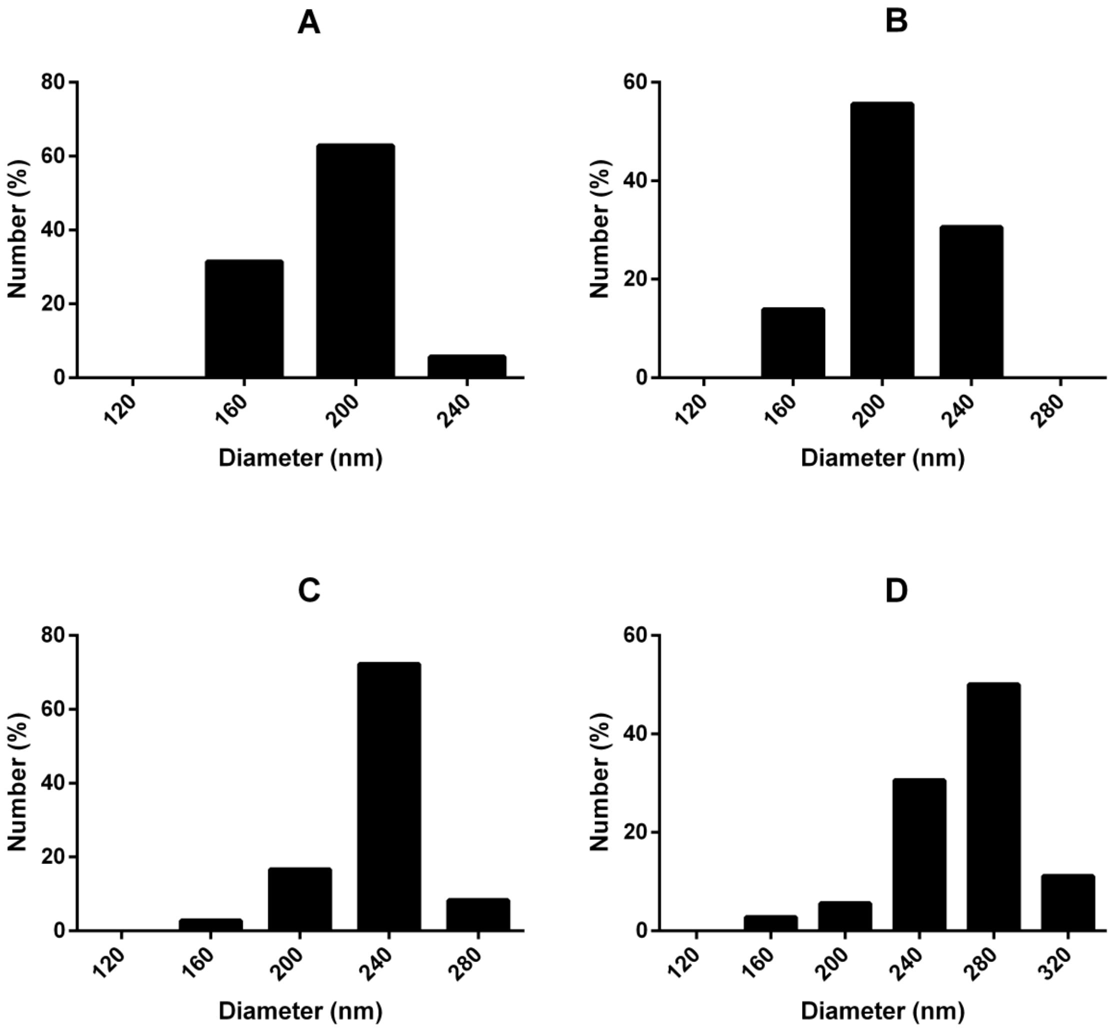

Figure S1

A

	Diameter (nm)	Z-potential (mV)
**SeNPs-48**	276±26	-29.27

B

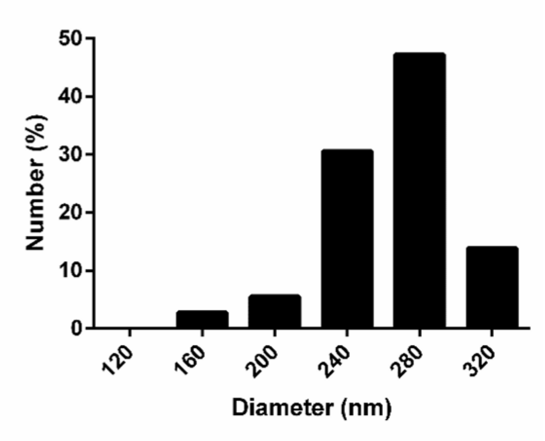

C

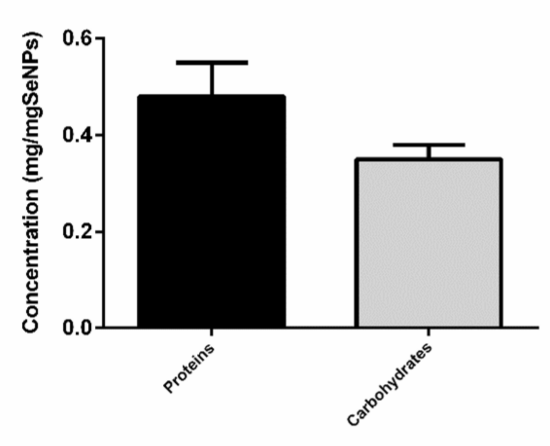

D

Bacterial Species	Strain Name	MIC µg/ml SeNPs-48
P. aeruginosa	PAO1	16
P. aeruginosa	BR2	32
S. maltophilia	VR20	256
S. aureus	UR1	16

Figure S2
